# Description of the first species of *Fiorianteon* Olmi (Hymenoptera, Dryinidae) from the Afrotropical region

**DOI:** 10.3897/zookeys.632.10576

**Published:** 2016-11-16

**Authors:** Adalgisa Guglielmino, Massimo Olmi, Alessandro Marletta, Stefano Speranza

**Affiliations:** 1 Department of Agriculture and Forestry Sciences (DAFNE), University of Tuscia, Viterbo, Italy; 2 Tropical Entomology Research Center, Viterbo, Italy; 3 Department of Biological, Geological and Environmental Sciences, Animal Biology section, University of Catania, Catania, Italy

**Keywords:** Taxonomy, Fiorianteon sulcatum, Madagascar, Conganteoninae, Chrysidoidea

## Abstract

*Fiorianteon sulcatum* **sp. n**. is described from Fianarantsoa Province (Madagascar). It is the first species of *Fiorianteon* found in the Afrotropical region. The genus *Fiorianteon* can be distinguished from the closely related genus *Conganteon* by the distal part of the stigmal vein, which is as long as, or shorter than the proximal part of the stigmal vein (longer than the proximal part of the vein in *Conganteon*).

## Introduction

Dryinidae (Hymenoptera Chrysidoidea) are parasitoids of Hemiptera, Auchenorrhyncha (Guglielmino et al. 2008, 2013). The biology of this small group of wasps is still poorly known (Carcupino et al. 1998; Guglielmino 2000; Guglielmino and Bückle 2003, 2010; Guglielmino et al. 2006, 2015; Guglielmino and Virla 1998).

The genus *Fiorianteon* Olmi, 1984 (Conganteoninae) is only present in the Oriental and Eastern Palaearctic zoogeographical regions (Olmi and Xu 2015). Four species have been described from the above regions (Xu et al. 2013; Olmi and Xu 2015). The hosts are unknown.

The genus was originally revised at world level by Olmi (1984) and more recently by Xu et al. (2013) and Olmi and Xu (2015) for the Oriental and the Eastern Palaearctic regions respectively.

In 2015, we examined additional specimens of Dryinidae from Madagascar, which included the new species of *Fiorianteon* described in this paper.

## Material and methods

The descriptions follow the terminology used by Olmi (1984), Olmi and Guglielmino (2010) and Olmi and Virla (2014). The reported measurements are relative, except for the total length (head to abdominal tip, without antennae), which is expressed in millimeters. In the descriptions, POL is the distance between the inner edges of the two lateral ocelli; OL is the distance between the inner edges of a lateral ocellus and the median ocellus; OOL is the distance from the outer edge of a lateral ocellus to the eye; OPL is the distance from the posterior edge of a lateral ocellus to the occipital carina; and TL is the distance from the posterior edge of an eye to the occipital carina. The material studied in this paper is deposited in the collections of the California Academy of Sciences, San Francisco, USA (CAS).

The multifocal pictures were taken by a stereomicroscope Leica M205A and Leica DFC450 video camera, captured using Leica Application Suite v. 4.2.0.

## Results

### Genus Fiorianteon

Taxon classificationAnimaliaHymenopteraDryinidae

Olmi, 1984

Fiorianteon Olmi, 1984: 108. Type species: Fiorianteon junonium Olmi, 1984, by original designation.

#### Diagnosis.

**Female**: fully winged; occipital carina complete; mandible quadridentate, with one intermediate rudimentary tooth; antenna without rhinaria; palpal formula 6/3; pronotal tubercles present; forewing with two cells enclosed by pigmented veins (costal and median); forewing with stigmal vein and pterostigma present; distal part of stigmal vein as long as, or shorter than proximal part of stigmal vein; protarsus chelate; chela with rudimentary claw; tibial spurs 1/1/2. **Male**: fully winged; occipital carina complete; mandible quadridentate, with one intermediate rudimentary tooth; palpal formula 6/3; forewing with two cells enclosed by pigmented veins (costal and median; fore wing with stigmal vein and pterostigma present; distal part of stigmal vein as long as, or shorter than proximal part of stigmal vein; tibial spurs 1/1/2.

### Fiorianteon sulcatum

Taxon classificationAnimaliaHymenopteraDryinidae

Guglielmino, Olmi, Marletta & Speranza sp. n.

http://zoobank.org/6D43414A-BCB9-4C75-BF6C-39D6498599B5

#### Diagnosis.

Head completely sculptured by longitudinal subparallel keels, on face (Fig. 3), vertex and temple; paramere (Fig. 4) with distal part of inner margin provisioned with many sensorial processes.

**Figures 1–3.**
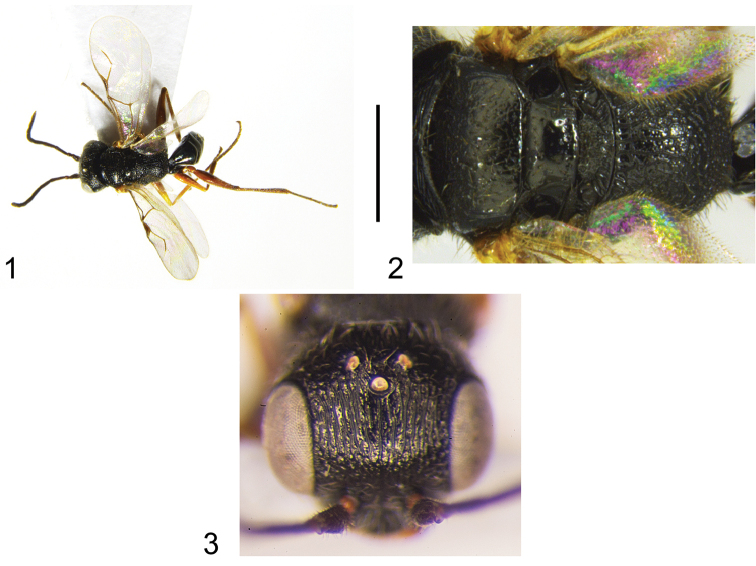
Male holotype of *Fiorianteon sulcatum* sp. n..: habitus (**1**) and mesosoma (**2**) in dorsal view; head in frontal view (**3**). Scale bar = 2.53 mm (**1**), 0.37 mm (**2**); 0.45 mm (**3**).

**Figure 4.**
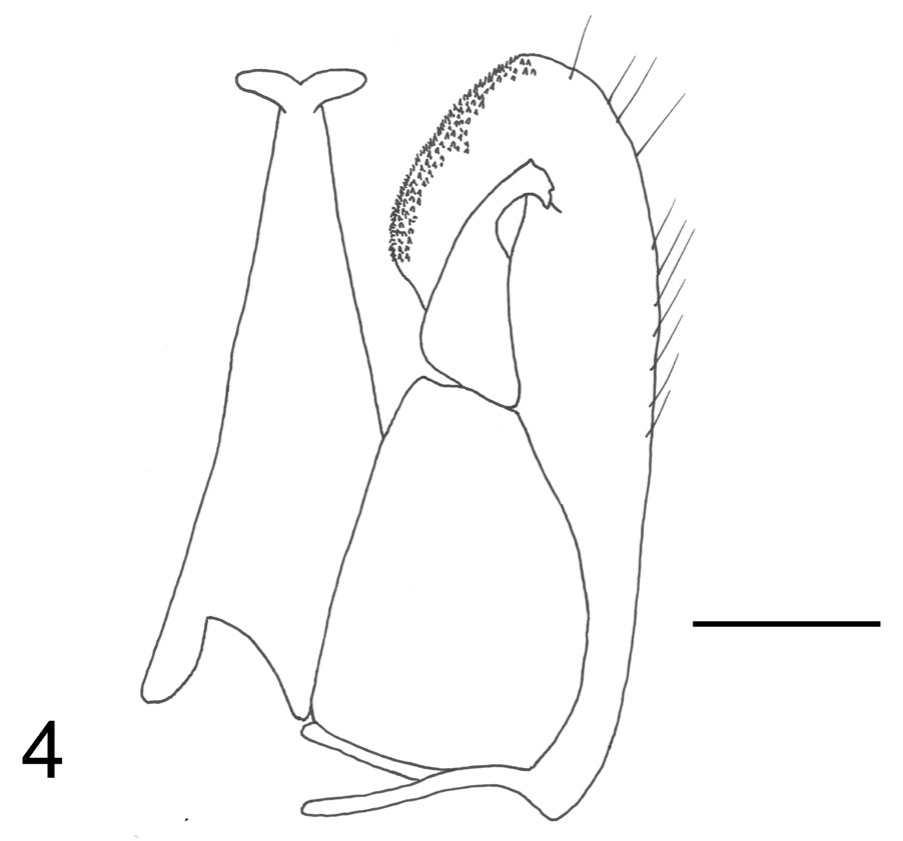
Male holotype of *Fiorianteon sulcatum* sp. n..: male genitalia (left half removed). Scale bar = 0.10 mm.

#### Description.

**Male.** Fully winged (Fig. 1). Body length 2.8 mm. Head black, except mandible testaceous; antenna brown; mesosoma and metasoma black; legs brown, except most part of coxae black. Antenna filiform; antennal segments in following proportions: 11:5:13:14:13:12:10:9:8:10. Head shiny, completely sculptured by longitudinal subparallel keels, on face (Fig. 3), vertex and temple; frontal line complete; occipital carina complete; POL = 5; OL = 3; OOL = 7; OPL = 7; TL = 10; greatest breadth of lateral ocelli about as long as OL. Scutum (Fig. 2) shiny, with anterior half slightly rugose; posterior half, punctate, unsculptured among punctures. Notauli incomplete, reaching approximately 0.5× length of scutum. Scutellum punctate, unsculptured among punctures. Metanotum dull, rugose. Propodeum reticulate rugose, without transverse or longitudinal keels. Forewing hyaline, without dark transverse bands; distal part of stigmal vein about as long as proximal part (Fig. 1), about as long as antennal segment 3. Paramere (Fig. 4) with distal part of inner margin provided of many sensorial processes. Tibial spurs 1/1/2.

**Female.** Unknown.

#### Material examined.

**Holotype**: male, MADAGASCAR: Fianarantsoa Province, Andringitra National Park, Plateau d’Andohariana, 35.9 km 205° Ambalavao, 22°09.08'S 46°53.57'E, 2000 m, 15.IV.2006, Malaise trap, BL Fisher et al. leg., BLF13755 (CAS).

#### Hosts.

Unknown.

#### Distribution.

Madagascar.

#### Remarks.

The two main characters distinguishing the new species are detailed in the above diagnosis. These characters are not present in any of the known species of Conganteoninae (Olmi and Xu 2015; Xu et al. 2013).

#### Etymology.

The species is named *sulcatum* because the head is sculptured by many longitudinal subparallel keels.

## Discussion

Azevedo et al. (2010) listed 123 species, 15 genera and 7 subfamilies of Dryinidae from the Malagasy region. The recorded genera and subfamilies were as follows: Anteoninae: *Anteon* Jurine, 1807 (28 species), *Deinodryinus* Perkins, 1907 (13 species), *Lonchodryinus* Kieffer, 1905 (three species); Aphelopinae: *Aphelopus* Dalman, 1823 (three species); Apodryininae: *Apogonatopus* Olmi, 2007 (two species), *Gondwanadryinus* Olmi, 2007 (one species), *Madecadryinus* Olmi, 2007 (six species); Bocchinae: *Bocchus* Ashmead, 1893 (eight species); Conganteoninae: *Conganteon* Benoit, 1951 (two species); Dryininae: *Dryinus* Latreille, 1804 (16 species), *Thaumatodryinus* Perkins, 1905 (six species); Gonatopodinae: *Echthrodelphax* Perkins, 1903 (two species), *Gonatopus* Ljungh, 1810 (30 species), *Haplogonatopus* Perkins, 1905 (one species) and *Neodryinus* Perkins, 1905 (two species). With the description of the above new species the number of species in the Malagasy region is elevated to 124 and the genera to 16.

## Supplementary Material

XML Treatment for Fiorianteon

XML Treatment for Fiorianteon sulcatum
